# Deepweed: automated individual segmentation and tracking for duckweed lineage analysis

**DOI:** 10.1093/bioinformatics/btag779

**Published:** 2026-10-16

**Authors:** Tolulope Oyeniran, Bukola Akindipe, Tasmia Islam, Joshua D Scheel, Eric A Josephs, Samuel M D Oliveira

**Affiliations:** Department of Nanoengineering, North Carolina Agricultural and Technical State University, Greensboro, NC 27401, United States; Department of Nanoengineering, North Carolina Agricultural and Technical State University, Greensboro, NC 27401, United States; Department of Nanoscience, University of North Carolina at Greensboro, Greensboro, NC 27403, United States; Department of Biomedical Engineering, University of Texas at Austin, Austin, TX 78712, United States; Department of Nanoscience, University of North Carolina at Greensboro, Greensboro, NC 27403, United States; Department of Biomedical Engineering, Stony Brook University, Stony Brook, NY 11794, United States; Department of Nanoengineering, North Carolina Agricultural and Technical State University, Greensboro, NC 27401, United States

## Abstract

**Motivation:**

Aquatic duckweeds, including *Wolffia australiana*, are the smallest and fastest growing flowering plants on the planet, with numerous emerging biotechnological applications. Duckweed populations grow through rapid clonal budding, but existing phenotypic characterization and image analysis tools typically operate only at the population level, tracking neither individual plants nor their lineage structure. Deepweed addresses this gap, combining automated individual segmentation, temporal tracking, and clonal lineage reconstruction from time-lapse imaging of duckweed.

**Results:**

Deepweed processes time-lapse duckweed imaging datasets using deep-learning–based instance segmentation and multi-object tracking to assign persistent identities to individual fronds and infer mother–daughter relationships. The workflow outputs lineage graphs, individual-level morphological metrics, and population-level growth summaries, supported by lineage-annotated time-lapse visualizations. Applied to dense *Wolffia* populations, in both freely growing or controlled microfluidic environments, our framework can track hundreds of *Wolffia* plants simultaneously, resolves overlapping individuals, and reconstructs multi-generation lineage structure, enabling analyses that are inaccessible with population-level phenotyping methods.

**Availability:**

Deepweed is implemented in Python using PyTorch, scikit-image, OpenCV, and btrack. Source code, trained models, example datasets, and documentation are freely available at https://github.com/SamOliveiraLab/Deepweed. The Deepweed code is archived at https://doi.org/10.5281/zenodo.18844282 and the datasets are archived at https://doi.org/10.5281/zenodo.18843917.

## 1 Introduction

Duckweeds are the smallest and fastest-growing angiosperms, with species such as *Wolffia australiana* having simplified morphologies consisting of a leaf-like “frond” measuring approximately 1 mm in diameter (Acosta *et al*. 2021). While capable of flowering, duckweeds reproduce almost exclusively through clonal budding of genetically identical daughter fronds. Doubling times vary substantially with species and clone: *Wolffia* doubles in under a day (Lam and Michael 2022), whereas other Lemnaceae require up to four days under standardized conditions (Ziegler *et al*. 2015). Their rapid growth, small size, and simplified genomes make duckweeds a valuable model for plant development, stress physiology, aquatic ecology, bioremediation, and biopharmaceutical production (Lam and Michael 2022), all of which require quantitative measurements of individual frond growth and budding.

However, despite increasing interest in duckweed phenotyping, most existing computational approaches quantify growth at the population scale, treating clusters of duckweed plants, often composed of a mother frond and multiple generations of daughters and grand-daughters in the process of emerging from one another, as a single object and producing only aggregate measurements such as total area, total frond count, or color indices (Cox *et al*. 2022, Kose *et al*. 2023, Subbaraman *et al*. 2024). Existing platforms, including time-lapse systems (Cox *et al*. 2022), high-throughput pipelines (Kose *et al*. 2023), workflow tools (Subbaraman *et al*. 2024), and frond detectors (Kurnia *et al*. 2023), cannot track individual plants over time or quantify intra-colony growth differences, perturbation effects on budding timing, or generational contributions to population expansion. This lack of lineage-resolved phenotyping limits developmental studies, constrains the interpretation of stress-response experiments, and restricts computational modeling of clonal plant systems (Fahlgren *et al*. 2015, Minervini *et al*. 2015).

We present Deepweed, a computational framework for individual frond tracking and mother-daughter lineage reconstruction from time-lapse imaging of both freely grown (hydroponic) and microfluidic ‘plant-on-chip’ (Feng Li *et al.* 2023) duckweed setups (Fig. 1A). The automated analysis workflow (Fig. 1B–E) combines deep-learning–based instance segmentation with multi-object tracking to assign persistent identities to individual fronds and to infer mother–daughter relationships over time. The system outputs lineage trees, per-frond morphological features, population-level growth curves, and generational summaries, enabling phenotypic resolution beyond that of population-level methods. By resolving the internal structure and dynamics of clonal growth, Deepweed delivers lineage-resolved phenotyping of *Wolffia*, including individual growth trajectories, budding timing, and generational contributions to population expansion, demonstrated in both free-floating and spatially confined culture.

**Figure 1:**
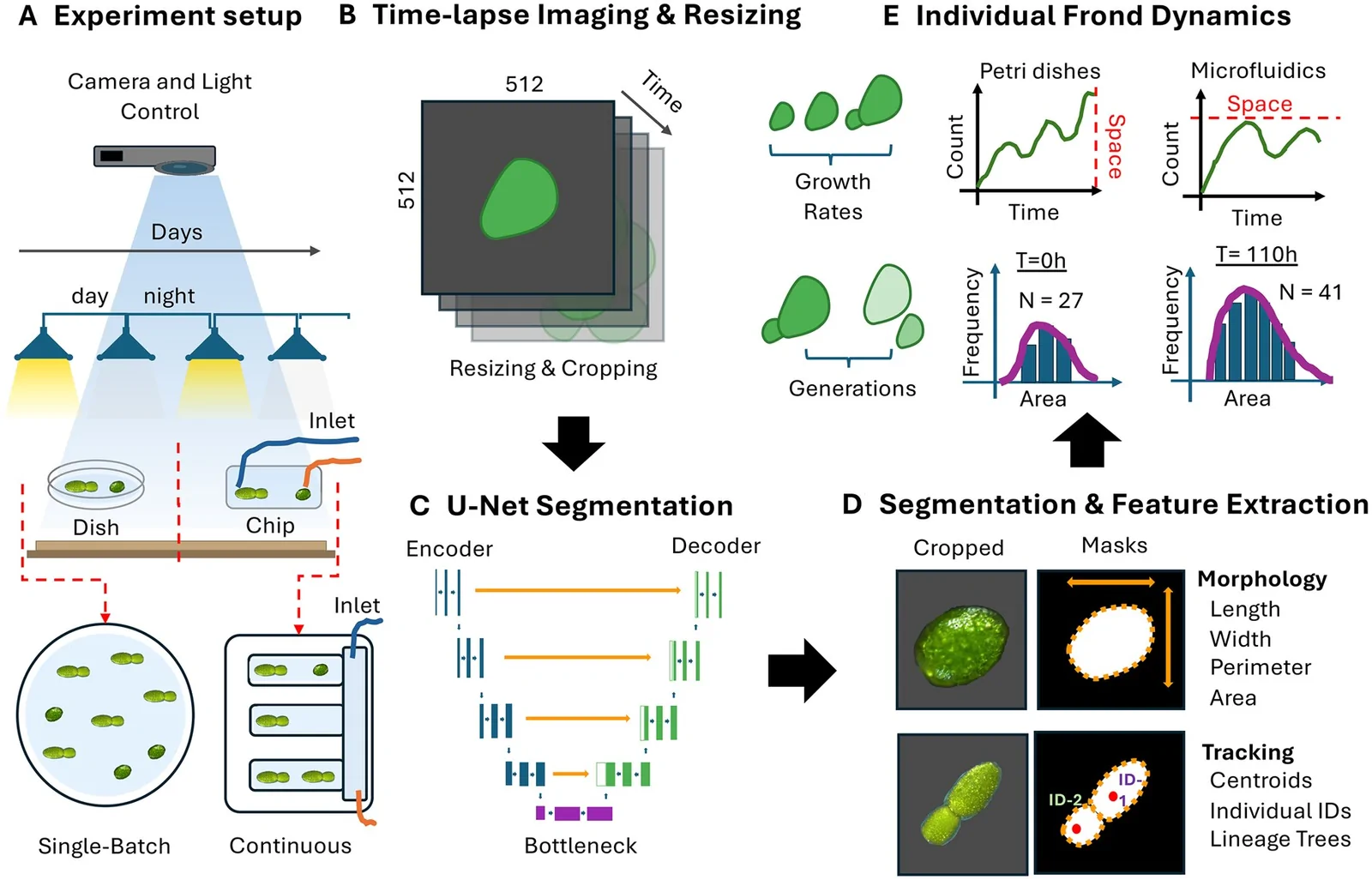
Deepweed workflow overview. (A) Experimental setup showing camera and light control for time-lapse imaging of duckweed in petri dishes or microfluidic chips. (B) Raw time-lapse frames are resized and cropped to 512 × 512 pixels. (C) U-Net encoder-decoder architecture performs instance segmentation. (D) Segmentation masks enable morphological feature extraction (length, width, perimeter, area) and tracking with persistent identity assignment (centroids, individual IDs, lineage trees). (E) Individual frond dynamics: growth rates, generational lineages, and population size distributions over time. Frond counts are bounded by the physical limits (‘Space’) of each platform, set by dish diameter in the petri dish and by trench length in the microfluidic chip.

## 2 Materials and methods

### 2.1 Image acquisition

Training and validation data were acquired from *Wolffia australiana* DWC304 collected in Australia (New South Wales, Singleton, Doughboy Hollow), obtained from the Rutgers Duckweed Stock Cooperative (RDSC), and grown under two experimental setups: (1) microfluidic cultivation chips for continuous-flow observation and (2) 12-well plate Petri dishes for free-floating batch cultures (Fig. 1A). Polydimethylsiloxane (PDMS) microfluidic devices were floated in liquid growth medium (General Hydroponics Flora Series, 1:1:1) in 120 mm polystyrene Petri plates. Single Wolffia plants were cultured under a full-spectrum LED grow light (400 mm above) with a 16 h/8 h light/dark photoperiod. We monitored plant growth using an LED digital microscope (USB2-MICRO-250X) connected to a laptop for image capture and documentation (Fig. S1, available as supplementary data at *Bioinformatics* online**).** We used the same liquid growth medium and cultivation conditions for cultures grown in 12-well plates. We acquired time-lapse images at 20-min intervals over 110 h. Hardware specifications and cultivation conditions are provided in Table S1, available as supplementary data at *Bioinformatics* online.

### 2.2. Segmentation model

Deepweed performs automated instance segmentation of duckweed leaf-like bodies (‘fronds’) using a U-Net encoder-decoder architecture with skip connections (Ronneberger *et al*. 2015), implemented in PyTorch (Fig. S2, available as supplementary data at *Bioinformatics* online). The model takes 512 × 512 RGB images as input and outputs segmentation masks of the same resolution. The petri dish model was originally trained at 256 × 256 and retrained at 512 × 512 to improve boundary fidelity at full image resolution (1600 × 1200).

#### 2.2.1 Training data

Separate models were trained per setup. The microfluidic model used 60 annotated images (50/10 train/val split); the Petri dish model used 80 annotated images (70/10 train/val split) drawn from time-lapse sequences like those shown in Fig. S3, available as supplementary data at *Bioinformatics* online. Ground-truth masks were created in Napari.

#### 2.2.2 Training configuration

Both models were trained with identical hyperparameters: 470 epochs, Dice loss, Adam optimizer with an initial learning rate of 1 × 10^−^^4^, ReduceLROnPlateau scheduler (factor = 0.5, patience = 20), dropout rate of 0.2, and batch size of 8. To improve generalization across varying imaging conditions, we applied on-the-fly data augmentation during training using the Albumentations library (Buslaev *et al*. 2020).

#### 2.2.3 Performance

The microfluidics model achieved a Dice coefficient of 0.6767 and IoU of 0.5166 on its validation set. For the petri dish model, we quantified performance on an independent test set of 10 frames acquired 24–59 hours after the final training frame, containing 324 annotated fronds and annotated without reference to model output, with no overlap with the training or model-selection data (Supplementary Methods Section 1.8, available as supplementary data at *Bioinformatics* online). Evaluated at full image resolution, the retrained 512 × 512 model achieved a body Dice coefficient of 0.908 +/− 0.006 and an IoU of 0.832 +/− 0.010, detecting 85.8% of fronds at 99.3% precision (Table S2, available as supplementary data at *Bioinformatics* online). This improves on the original 256 × 256 model (Dice 0.865, IoU 0.762, recall 77.5%), confirming that higher input resolution improves segmentation at full image scale. Representative segmentation outputs are shown in Fig. S4, available as supplementary data at *Bioinformatics* online. Retraining each model on random subsets of its training pool (Supplementary Methods Section 1.10, Fig. S5, available as supplementary data at *Bioinformatics* online) shows petri dish accuracy saturating at 50 training images (Dice 0.914) and unchanged at 70, while the microfluidic model improves from 0.404 to 0.563 across the same range.

#### 2.2.4 Inference

After training, the models generated a per-frame binary mask from raw video input. Connected component analysis then assigns unique instance labels to individual fronds, forming the foundation for downstream feature extraction and lineage tracking.

### 2.3. Morphological feature extraction

From each segmented frond instance, Deepweed extracts spatial coordinates (centroid, bounding boxes) and morphological features (area, perimeter, and major and minor axis lengths) on a per-frame basis. These measurements quantify frond size and shape at each time point. Temporal aggregation of these features enables calculation of growth rates, size distributions, and structural changes across the population over time.

### 2.4. Lineage tracking

Temporal correspondence of fronds across frames is established using btrack (Ulicna *et al*. 2021), a Bayesian tracking algorithm that links instances based on spatial proximity within a maximum search radius of 50 pixels (Table S3, available as supplementary data at *Bioinformatics* online). This tracking enables identification of mother-daughter relationships during reproduction events, reconstruction of complete lineage trees, and quantification of generational dynamics and population growth patterns. We infer mother–daughter relationships when a new instance appears within the defined search radius of an existing tracked frond and persists across subsequent frames. We assign each daughter to the generation following its parent, enabling multi-generational lineage reconstruction. *Limitations* of the tracking model are described in Supplementary Methods Section 1.7, available as supplementary data at *Bioinformatics* online.

### 2.5. Input data and processing pipeline

Deepweed accepts time-lapse microscopy videos of duckweed as input. The pipeline decomposes videos into individual frames, resizes them, and passes them to the segmentation model. The pipeline processes segmented instances per frame and establishes temporal links during tracking. The pipeline is implemented in Python using PyTorch for deep-learning inference, scikit-image for image processing, and OpenCV for video handling, with btrack providing temporal correspondence across frames.

### 2.6. Comparison with existing systems

We compared Deepweed with existing duckweed analysis tools. Prior work has developed automated imaging systems for time-lapse growth measurement (Cox *et al*. 2022), high-throughput phenotyping platforms for large-scale experiments (Kose *et al*. 2023), laboratory workflow management software (Scott *et al*. 2023), and robotic sample handling systems (Subbaraman *et al*. 2024). Our segmentation and tracking pipeline builds on general-purpose individual-object image analysis approaches such as SCIP, originally developed for single-cell microscopy (Martins *et al*. 2018), adapted here to multicellular duckweed fronds. However, these systems operate at the population level, quantifying aggregate metrics such as colony-wide growth rates, e.g. measured by bulk optical density or integrated fluorescence signals. None provides individual frond identification, persistent tracking across frames, or lineage reconstruction. Deepweed addresses this gap by integrating instance segmentation with temporal tracking to enable individual-resolved phenotyping and mother–daughter lineage inference.

## 3 Results

Deepweed successfully segmented duckweed leaf-like bodies (‘fronds’) in time-lapse videos acquired in microfluidic chips and Petri dish setups. We selected the microfluidic chip model from epoch 117, corresponding to the best validation performance (Dice = 0.6767; Table S2; Fig. S6, available as supplementary data at *Bioinformatics* online); the Petri dish model achieved Dice = 0.9420. Visual inspection confirmed that the model accurately delineated frond boundaries across frames, including partially overlapping and variably illuminated fronds.

Deepweed extracted per-frond morphological descriptors (area, perimeter, axis lengths) to generate continuous growth trajectories (Fig. S7, available as supplementary data at *Bioinformatics* online), revealing cyclic area changes in mother fronds and steady post-separation expansion in daughters.

These single-frond dynamics revealed characteristic growth patterns, with mother fronds showing cyclic area changes associated with daughter production and daughter fronds exhibiting steady expansion following separation (Fig. S7, available as supplementary data at *Bioinformatics* online). Per-generation size distributions showed a decrease in mean area from Gen 0 (0.030 mm^2^) to Gen 2 (0.016 mm^2^), consistent with successive budding events (Fig. S8; Table S4, available as supplementary data at *Bioinformatics* online).

Using btrack, Deepweed linked segmented fronds across frames to recover multigenerational lineages (Fig. S9, available as supplementary data at *Bioinformatics* online), with mother–daughter assignments consistent with manual curation and lineage trees capturing expected clonal branching behavior (Fig. S10; Table S5, available as supplementary data at *Bioinformatics* online).

In a representative experiment spanning 4.7 days (338 frames at 20-minute intervals) and 192 unique tracked fronds, the complete Deepweed pipeline processed 12 824 individual detections and identified 24 division events across 2 generations (Table S3, available as supplementary data at *Bioinformatics* online). To quantify tracking accuracy, we computed identity switch rates by measuring how often a frond’s track assignment changed between consecutive frames (Supplementary Methods Section 1.9, available as supplementary data at *Bioinformatics* online). In the microfluidic chip experiment, the ID switch rate was 0.57% (78 switches across 13 757 frame-to-frame associations); in the Petri dish experiment (1664 frames, 138.7 hours), the rate was 0.11% (35 switches across 32 111 associations). Partitioning each experiment into temporal quarters showed no systematic increase in error rate over time (Table S3, available as supplementary data at *Bioinformatics* online). Population growth followed an exponential (Fig. S11; Table S6, available as supplementary data at *Bioinformatics* online). Track fragmentation, where a single frond’s trajectory is split into multiple track segments, affected 10.1% of microfluidic tracks and 11.7% of Petri dish tracks; a post-hoc stitching step merged these fragments based on spatiotemporal proximity, and an interactive review tool (Fig. S12, available as supplementary data at *Bioinformatics* online) allowed manual confirmation of individual track joins. Tracking error rises predictably with acquisition interval, a general property of btrack (Fig. S13, available as supplementary data at *Bioinformatics* online). The analysis required approximately 30 minutes on a workstation equipped with an MPS GPU, demonstrating suitability for routine analysis of microfluidic duckweed experiments.

The longer chip doubling time likely reflects non-budding plants constrained by channel geometry; we are refining the analysis to separate budding from non-budding lineages. The analysis required approximately 30 minutes on a GPU-accelerated workstation, demonstrating suitability for routine analysis of duckweed experiments. Example time series are presented in Supplementary Videos S1–S4, available as supplementary data at *Bioinformatics* online.

## 4 Discussions

Deepweed combines individual frond segmentation, temporal tracking, and mother–daughter relationship inference to reconstruct growth and clonal lineages in *Wolffia australiana*. Across Petri dish and microfluidic cultures, the workflow captured mother-frond area cycles, daughter expansion, and multigenerational relationships (Figs. S7–S10, available as supplementary data at *Bioinformatics* online). By linking morphological measurements to persistent identities and ancestry, Deepweed connects population growth to individual plants’ developmental histories.

Several limitations define the current implementation. We trained separate models for each culture setup, with differing segmentation performance (Table S2, available as supplementary data at *Bioinformatics* online), and transfer beyond *W. australiana* requires validation and potentially additional annotations. Dense clusters reduce segmentation recall and limit lineage completeness (Fig. S14, available as supplementary data at *Bioinformatics* online), while longer acquisition intervals increase tracking errors (Fig. S13, available as supplementary data at *Bioinformatics* online). Next, while track stitching and interactive review help resolve fragmentation, ambiguous trajectories and mother–daughter assignments still require inspection (Fig. S12, Table S3, available as supplementary data at *Bioinformatics* online). Moreover, projected area does not directly measure biomass, and differences between generations may reflect their age distributions. Finally, microfluidic confinement may also influence growth and budding, and distinguishing these effects from intrinsic biological differences requires controlled comparisons.

Within these limits, Deepweed enables questions that bulk-population analysis alone cannot resolve. For example, comparing observable budding events with light–dark cycles could clarify the relationship between reproductive timing and the reduced temporal regulation associated with rapid growth in Wolffia (Lam and Michael 2022). In stress and toxicity assays (Kurnia *et al*. 2023), individual trajectories could distinguish reduced frond expansion from delayed or less frequent budding, while lineage information could reveal whether responses differ among families or generations. For biotechnology, these measurements could help compare cultivation conditions by identifying their effects on individual expansion and daughter production. Nevertheless, growth trajectories and budding intervals could also inform models linking individual development to population dynamics.

Deepweed extends existing duckweed imaging approaches. For instance, automated microscopy has enabled plantlet growth measurements (Cox *et al*. 2022), while high-throughput imaging and experimental automation have expanded the scale of growth assays (Kose *et al*. 2023, Subbaraman *et al*. 2024). In addition, deep-learning methods have also improved frond detection and counting (Kurnia *et al*. 2023). However, Deepweed enables maintaining identities over time and connects individuals through inferred ancestry. It does so by building on Bayesian methods for cell tracking and lineage reconstruction (Ulicna *et al*. 2021) and applying this approach to whole clonal plants, supporting duckweed’s development as a model for plant and synthetic biology (Acosta *et al*. 2021, Lam and Michael 2022).

More broadly, clonal populations provide an opportunity to investigate how developmental history and environmental experience generate variation against a largely shared genetic background. Linking growth and reproduction to ancestry can make this variation accessible to quantitative analysis. Deepweed therefore provides a foundation for understanding how individual plant histories can shape a clonal population’s growth and responses.

## Supplementary Material

btag779_Supplementary_Data

## References

[btag779-B1] Acosta K , Appenroth KJ, Borisjuk L et al Return of the Lemnaceae: duckweed as a model plant system in the genomics and postgenomics era. Plant Cell 2021;33:3207–34. 10.1093/plcell/koab189 PMC8505876 34273173

[btag779-B3] Buslaev A , Iglovikov VI, Khvedchenya E et al Albumentations: fast and flexible image augmentations. Information 2020;11:125. 10.3390/info11020125

[btag779-B4] Cox KL Jr , Manchego J, Meyers BC et al Automated imaging of duckweed growth and development. Plant Direct 2022;6:e439. 10.1002/pld3.439 PMC9510441 36186894

[btag779-B5] Fahlgren N , Gehan MA, Baxter I. Lights, camera, action: high-throughput plant phenotyping is ready for a close-up. Curr Opin Plant Biol 2015;24:93–9. 10.1016/j.pbi.2015.02.006 25733069

[btag779-B6] Feng Li J-J , Yang Z-Y, Sun L et al Plant-on-chip: core morphogenesis processes in the tiny plant *Wolffia australiana*. PNAS Nexus 2023;2:pgad141. 10.1093/pnasnexus/pgad141 PMC10169700 37181047

[btag779-B7] Kose T , Lins TF, Wang J et al Accelerated high-throughput imaging and phenotyping system for small organisms. PLoS One 2023;18:e0287739. 10.1371/journal.pone.0287739 PMC10361482 37478145

[btag779-B8] Kurnia KA , Lin Y-T, Farhan A et al Deep learning-based automatic duckweed counting using StarDist and its application on measuring growth inhibition potential of rare earth elements as contaminants of emerging concerns. Toxics 2023;11:680. 10.3390/toxics11080680 PMC10457735 37624185

[btag779-B9] Lam E , Michael TP. Wolffia, a minimalist plant and synthetic biology chassis. Trends Plant Sci 2022;27:430–9. 10.1016/j.tplants.2021.11.014 34920947

[btag779-B10] Martins L , Neeli-Venkata R, Oliveira SMD et al SCIP: a single-cell image processor toolbox. Bioinformatics 2018;34:4318–20. 10.1093/bioinformatics/bty505 29931314

[btag779-B11] Minervini M , Scharr H, Tsaftaris SA. Image analysis: the new bottleneck in plant phenotyping. IEEE Signal Process Mag 2015;32:126–31. 10.1109/MSP.2015.2405111

[btag779-B12] Ronneberger O , Ronneberger O, Fischer P et al U-net: Convolutional networks for biomedical image segmentation. In: Medical Image Computing and Computer-Assisted Intervention – MICCAI 2015. Lecture Notes in Computer Science. vol 9351. Cham: Springer, 2015. 10.1007/978-3-319-24574-4_28

[btag779-B13] Scott M , de Lange O, Quaranto X et al Open-source workflow design and management software to interrogate duckweed growth conditions and stress responses. Plant Methods 2023;19:95. 10.1186/s13007-023-01065-3 PMC10472582 37653538

[btag779-B14] Subbaraman B , de Lange O, Ferguson S et al The Duckbot: a system for automated imaging and manipulation of duckweed. PLoS One 2024;19:e0296717. 10.1371/journal.pone.0296717 PMC10805289 38261570

[btag779-B15] Ulicna K , Vallardi G, Charras G et al Automated deep lineage tree analysis using a Bayesian single cell tracking approach. Front Comput Sci 2021;3:734559. 10.3389/fcomp.2021.734559

[btag779-B16] Ziegler P , Adelmann K, Zimmer S et al Relative in vitro growth rates of duckweeds (Lemnaceae): the most rapidly growing higher plants. Plant Biol (Stuttg) 2015;17:33–41. 10.1111/plb.12184 24803032

