# Supplementary material for: Deepweed: automated individual segmentation and tracking for duckweed lineage analysis
Source: Bioinformatics. 2026 Oct 16;42(10):btag779. doi: 10.1093/bioinformatics/btag779 (PMC13713196; doi:10.1093/bioinformatics/btag779)
Supplement: Supplementary file 1 [file btag779_supplementary_data.zip › Oliveira2026_SI_Final.docx]

**Supplementary Information for** “Deepweed: Automated Individual Segmentation and Tracking for Duckweed Lineage Analysis”

Tolulope Oyeniran^1,+^, Bukola Akindipe^1,+^, Tasmia Islam^2^, Joshua D. Scheel^4^, Eric A. Josephs^2,3^, and Samuel M.D. Oliveira^1,*^

^1^Department of Nanoengineering, North Carolina Agricultural and Technical State University, Greensboro, NC, USA 27401,^2^Department of Nanoscience, University of North Carolina at Greensboro, NC, USA 27403,^3^Department of Biomedical Engineering, Stony Brook University, 100 Nicolls Road, Stony Brook, NY, USA 11794, ^4^Department of Biomedical Engineering, University of Texas at Austin, Austin, TX, USA 78712

⁺Authors contributed equally

*Corresponding author: [smdoliveira@ncat.edu](mailto:smdoliveira@ncat.edu)

Table of Contents

[1. Supplementary Methods	2](#_Toc238147207)

[1.1. Experimental Setup and Image Acquisition	2](#_Toc238147208)

[1.2. Image Preprocessing	2](#_Toc238147209)

[1.3. Deep Learning Model Architecture and Training	2](#_Toc238147210)

[1.4. Segmentation and Morphological Feature Extraction	2](#_Toc238147211)

[1.5. Temporal Tracking and Lineage Reconstruction	3](#_Toc238147212)

[1.6. Population Growth Analysis	3](#_Toc238147213)

[1.7. Lineage Tracking Limitations	3](#_Toc238147214)

[1.8. Independent Test Set Evaluation	3](#_Toc238147215)

[1.9. Tracking Error Metrics	3](#_Toc238147216)

[1.10. Training Set Size Sensitivity	4](#_Toc238147217)

[2. Supplementary Figures	5](#_Toc238147218)

[3. Supplementary Tables	19](#_Toc238147219)

[4. Supplementary Data	25](#_Toc238147220)

[5. Supplementary Videos	26](#_Toc238147221)

# 1. Supplementary Methods

## 1.1. Experimental Setup and Image Acquisition

Duckweed fronds (*Wolffia australiana* DWC304, Australia (New South Wales, Singleton, Doughboy Hollow) obtained from the Rutgers Duck-weed Stock Cooperative (RDSC)) were cultured in 12-well multi-well plate or a custom microfluidic chip consisting of 28 channels that were 0.5 mm in width and 8 mm in length Figure S3) floating atop growth solution in a 120 mm × 15 mm petri dish designed for long-term time-lapse imaging under controlled hydroponic conditions. In both cases, each culture started from a single frond. The microfluidic system provides spatial confinement to maintain fronds within the imaging field of view, controlled nutrient delivery, and optical access for high-quality brightfield microscopy.

Time-lapse images were acquired via a USB-pluggable digital microscope camera (250x, 2MP) at 20-minute intervals over 112.7 hours (approximately 4.7 days), yielding 338 frames. Images were captured as individual JPEG files with timestamp information encoded in filenames. For visualization and quality control, individual frames were subsequently compiled into video format (Supplementary Videos S1-4); however, all quantitative analyses were performed on the original JPEG frames. **Table S1** provides complete imaging specifications.

## 1.2. Image Preprocessing

Images were resized to 512 × 512 pixels using bilinear interpolation to accommodate the U-Net architecture requirements. Pixel intensities were normalized to [0, 1] by dividing by 255. Calibration factors for converting pixel measurements to physical units were empirically determined using objects of known dimensions: 0.00111 mm²/pixel for area and 0.03333 mm/pixel for length measurements (**Table S1**).

## 1.3. Deep Learning Model Architecture and Training

We employed a U-Net convolutional neural network for the semantic segmentation of duckweed fronds. The architecture comprises an encoder pathway with progressive *downsampling* and increasing feature channels, a bottleneck layer, and a decoder pathway with up-sampling and skip connections that preserve spatial information (Figure S1). The network accepts RGB input (512 × 512 × 3) and produces single-channel probability maps (512 × 512 × 1).

Training data consisted of manually annotated image-mask pairs with precise frond boundary delineation. Data augmentation was applied during training using the *Albumentations* library, including geometric transformations (rotation, flipping, elastic deformation) and photometric perturbations (brightness, contrast, HSV adjustments).

The model was trained for 470 epochs using the Adam optimizer with an initial learning rate of 1 × 10⁻⁴ and a ReduceLROnPlateau scheduler (factor = 0.5, patience = 20 epochs). Dice loss was used as the optimization objective. Dropout (rate=0.2) was applied after the first convolutional layer in each encoder block to regularize the model. The best model achieved a validation Dice coefficient of 0.6767 (IoU = 0.5166) at epoch 117 (**Figure S2**, **Table S2**).

## 1.4. Segmentation and Morphological Feature Extraction

The trained model was applied to all 338 frames to generate binary segmentation masks (probability threshold = 0.5). We achieved instance segmentation by applying connected component analysis to the binary masks, using watershed segmentation to separate touching fronds. Size filtering removed spurious detections with areas below a minimum threshold.

For each detected frond instance, we extracted morphological features, including area (mm²), perimeter (mm), and major- and minor-axis lengths (mm). Figure S7 shows representative segmentation results demonstrating model performance under different imaging conditions.

## 1.5. Temporal Tracking and Lineage Reconstruction

Temporal correspondence of segmented fronds across frames was established using the btrack Bayesian tracking algorithm with a maximum search radius of 50 pixels. The algorithm links detections based on spatial proximity, morphological similarity, and motion coherence.

Budding events were identified when new tracks appeared in immediate spatial proximity to existing tracks (typically <1 pixel distance, Table S6), consistent with daughter fronds emerging from mother fronds during vegetative reproduction. Each track was assigned a generation number: Generation 0 for founding fronds present at experiment initiation, and subsequent generations for offspring identified through budding detection. Table Statistics provides complete tracking statistics.

## 1.6. Population Growth Analysis

Population growth analysis quantified changes in individual numbers over time. We modeled growth dynamics using an exponential function, N(t) = N0 × exp(rt), where N(t) denotes population size at time t, N0 is the initial population, and r is the population growth rate. To reduce frame-to-frame noise from detection variability, we applied a 15-frame rolling average (5-hour window) before fitting the model. Parameters were estimated using non-linear least squares optimization (SciPy. optimize. curve_fit).

Morphological statistics were computed separately for each generation to assess size distributions across the lineage structure (Figure S7, Table S5).

## 1.7. Lineage Tracking Limitations

The automated tracking system occasionally fragments a single plant's trajectory into multiple track IDs, particularly when segmentation temporarily fails due to occlusion, focus drift, or overlap between neighboring fronds. While a multi-pass stitching algorithm chains most fragments back into continuous trajectories using spatial proximity and area similarity, some fragmentations remain - visible as color changes in the trajectory plots. We identified these cases through a post hoc diagnostic analysis and confirmed they do not represent distinct biological entities. The raw crop images at each timepoint provide visual ground truth for verifying track continuity. Future work will incorporate appearance-based re-identification to reduce fragmentation further.

## 1.8. Independent Test Set Evaluation

We quantified segmentation performance on an independent test set drawn from the same time-lapse but disjoint from all data used for training and model selection. The validation frames used during training are separated from the nearest training frame by a median of 15 minutes, which is short relative to the 24- to 72-hour doubling time of *Wolffia australiana*; they therefore depict the same individuals in essentially unchanged positions and do not constitute an independent sample. The independent test set comprises 10 frames sampled from the 419 frames acquired at least 24 hours after the final training frame, spanning 24 to 59 hours after the end of training and containing 324 annotated fronds. Ground-truth masks were drawn from a blank canvas without reference to model predictions, since seeding annotation with model output causes undetected fronds to be omitted from the ground truth and inflates apparent recall. All metrics are computed at full image resolution (1600 x 1200), not at the network input size.

## 1.9. Tracking Error Metrics

Identity (ID) switch rate measures how often a frond's track assignment changes between consecutive frames, computed as the fraction of frame-to-frame associations in which spatially matched detections carry different track identifiers. Track fragmentation rate measures the fraction of raw btrack tracks that continue an existing frond rather than starting a new individual, identified by spatiotemporal proximity of track endpoints (within 20 pixels and 3 frames). To assess whether errors accumulate over time, we partitioned each experiment into temporal quarters and computed the ID switch rate independently for each quarter. Neither experiment showed a systematic increase, indicating that tracking accuracy is maintained throughout the observation period. Supplementary videos are rendered with post-stitching labels using a display criterion of 12 pixels across detection gaps of up to 10 hours, reflecting the sessile nature of the fronds; tracks shorter than 15 frames are left unlabelled. Global stage displacements in the raw time-lapse (e.g., plate handling events) are detected by phase-correlation registration of consecutive frames, and track endpoints are matched in drift-corrected coordinates so that stage displacement alone does not break frond identities; where handling physically rearranges the fronds, new identities are assigned.

## 1.10. Training Set Size Sensitivity

Each model was retrained from scratch on random subsets of its training pool, in increments of 10 images with three random draws per size, then scored on held-out frames at full image resolution. Values are the mean and standard deviation across the three draws.

## 1.11. Acquisition-Interval Error Analysis

To characterize how tracking error scales with time between frames, we compared btrack's full-frame-rate output against subsampled sequences skipping every *k* frames, simulating longer acquisition intervals. We scored each subsampled run against the full-rate track set using a MOTA-based excess error rate (identity switches plus false negatives and false positives, normalized by ground-truth observations), the metric used to validate btrack in its original publication (Ulicna et al., 2021). We performed this on an independent cell-tracking reference dataset and on our own petri-dish data; for each, we fit excess error rate vs. acquisition interval with an exponential through the origin (**Figure S12**).

## 1.12. Interactive Track-Fragmentation Review Tool

To make residual track fragmentation (Section 1.9) directly auditable, we built an interactive review tool. For each candidate track break, the tool presents the frond where a track ends, the candidate frond where the next begins, and the same well zoomed out with both track identifiers shown, allowing a reviewer to confirm or reject the join. We used confirmed joins to manually audit the fragmentation rate reported in **Table S3** (**Figure S13**).

## 1.13. External and In-Domain Crowding Assessment

We assessed segmentation performance under increasing frond density using three hand-annotated test sets spanning a real density gradient. Two were drawn from an external, independent-lab Wolffia globosa growth-inhibition assay (Day 0 and Day 7 of exposure; 24 and 126 fronds, blank-canvas annotation), evaluated with the microfluidics model after recalibrating its confidence threshold for this domain via leave-one-out validation (threshold chosen from two annotated frames, scored on the third, held-out frame). The third was a genuinely dense, in-domain frame from this study's own petri-dish imaging rig (261 fronds, 55.3% coverage), evaluated with the 512x512 petri-dish model after flat-field illumination correction. The code release includes all three test sets and evaluation scripts for reproducibility.

# 2. Supplementary Figures

**Figure S1. Experimental setup for Imaging.** Imaging Station used for monitoring *Wolffia* growth in PDMS microfluidic devices. The imaging station consists of two adjustable LED digital microscopes (USB2-MICRO-250X) mounted on articulating arms and positioned above the benchtop workspace, with a desk lamp providing supplemental illumination for image capture.

**Figure S2. U-Net Model Architecture.** Schematic of the U-Net architecture used for duckweed segmentation. The encoder pathway (left) progressively down-samples the input images while increasing the number of feature channels. The decoder pathway (right) up-samples feature maps and incorporates skip connections from the corresponding encoder layers. Input: RGB images (512 × 512 × 3). Output: single-channel probability maps (512 × 512 × 1). Dropout (rate=0.2) is applied in encoder blocks for regularization.

**Figure S3. Representative input data from both cultivation platforms.** Raw frames shown as acquired, without preprocessing. Top row: microfluidic device at t = 0, 35, 75, and 112 h, acquired at 20-min intervals, cropped to a group of trenches. Bottom row: petri dish at t = 0, 45, 90, and 140 h, acquired at 5-min intervals, cropped to a single well. The two platforms were imaged in separate experiments, and timepoints are not matched between rows.

**Figure S4. Segmentation Performance Across Experimental Platforms.** (A) Camera image showing a portion of a PDMS microfluidic chip consisting of 28 channels that were 0.5 mm in width and 8 mm in length floating atop growth solution in a 120 mm × 15 mm Petri dish: Raw image, ground truth mask, U-Net prediction (Dice = 0.6767), and previous color-based approach for comparison. The deep learning model demonstrates robust performance across varying illumination conditions. (B) Petri dish: Raw image showing duckweed in a 6-well plate, ground truth mask, and U-Net prediction. The Petri dish model successfully generalizes to different imaging conditions, background complexity, and frond sizes.

**Figure S5. Segmentation accuracy against training set size.** (A) Petri dish. Top: three held-out test images with ground truth outlined in green; the yellow box marks the region enlarged below, where the published model's prediction is added in red. Bottom: Dice (blue) and IoU (red) against the number of annotated training images; each point is the mean of three models retrained from scratch on independent random draws, with the shaded band showing their spread. Dashed lines mark the published model on the same frames. Accuracy saturates at 50 training images and is unchanged at 70. (B) Microfluidic device. Panels as in (A). Accuracy improves steadily with training set size and reaches the published model at the full training set of 50 images. All scores are computed at full image resolution.

**Figure S6. Model Training and Validation Performance.** Training dynamics of the U-Net segmentation model. (A) Validation Dice coefficient over 470 training epochs, reaching a best value of 0.8720 (red star). (B) Learning rate schedule showing ReduceLROnPlateau adjustments. Initial learning rate: 1 × 10⁻⁴; final learning rate: ~1.3 × 10⁻⁵. (C) IoU and cross-correlation metrics over training epochs.

**Figure S7. Individual Frond Growth Trajectories.** (A) Microfluidic chip: Single-trench analysis. Area trajectories for fronds within a single linear trench (trench 13) over 112.7 hours. Gray shading indicates night periods. (B) Petri dish: Single-cluster analysis. Area trajectories for all tracked fronds within a single cluster over 25 hours. Gray shading indicates the night period. Frond areas are larger (1–4 mm²) compared to microfluidic experiments (0.01–0.05 mm²) due to different imaging magnification and calibration. Occasional color changes within a single trajectory (e.g., panel B, ~110 h) reflect track ID reassignment by the automated tracking algorithm, not the appearance of a new plant (See section SI-1.7 on Limitations). Visual continuity can be confirmed by the crop images shown above the plot (displaying the same plant during imaging transition).

**Figure S8. Size Distribution by Generation.** A) Microfluidic chip: Gen 0 (N = 161; mean = 0.06 mm²), Gen 1 (N = 92; mean = 0.05 mm²), Gen 2 (N = 15; mean = 0.04 mm²). (B) Petri dish: Gen 0 (N = 162; mean = 2.68 mm²), Gen 1 (N = 63; mean = 1.45 mm²), Gen 2 (N = 4; mean = 3.23mm²), Gen 3 (N = 7; mean =0.67mm2). Red curves: fitted distributions. Note: absolute area differences between platforms reflect different pixel calibration factors; relative generational patterns are comparable. These values represent time-averaged morphological snapshots across each generation. A more detailed morphological growth analysis will be presented in a separate study.

**Figure S9.** **Spatial Tracking Visualization.** (A) Microfluidic chip: 140 tracks maintained across 338 frames. Fronds show limited lateral movement due to chamber confinement. (B) Petri dish: 24 tracks maintained across 300 frames. Fronds exhibit greater spatial movement in the unconstrained environment. Circles indicate track start positions; squares indicate track end positions. Each color represents a unique frond identity maintained by the btrack algorithm.

**Figure S10. Lineage tree reconstruction and inheritance relationships.** (A) Microfluidic chip: Single-trench analysis. 24 budding events detected across 3 generations (Gen 0, 1, 2) within a single linear trench. Blue nodes represent individual frond tracks; connecting lines indicate parent-child relationships. (B) Petri dish: Single-cluster analysis. 5 budding events detected across 3 generations within a single cluster. Green nodes represent frond tracks. Both platforms successfully reconstruct multi-generational lineage structure, demonstrating the workflow's ability to track clonal reproduction dynamics.

**Figure S11. Population Growth Dynamics.** (A) Microfluidic chip: Single-trench analysis. Population of a single linear trench increased from 1 to 2 fronds over 112.7 hours. Doubling time = 80.3h. (B) Petri dish: Single-cluster analysis. Population of a single cluster increased from 2 to 7 fronds over 140 hours. Doubling time = 85.9h. Light green: raw frame-by-frame counts; dark green: 5-hour rolling average. The faster growth rate in Petri dishes likely reflects less spatial constraint compared to microfluidic chambers.

**Figure S12. Tracking error vs. acquisition interval.** Excess error rate (MOTA-based) for btrack, the algorithm used throughout this study, on an independent cell-tracking reference dataset (blue) and on this study's own petri-dish duckweed tracking (red), each scored against its own full-frame-rate reference. Dashed lines: exponential fits through the origin, R² per fit. Duckweed degrades far more slowly, consistent with the absence of division-driven identity ambiguity in cell tracking.

**Figure S13. Interactive correction of frond track fragmentation.** (A) Three candidate pairs as the tool presents them: the frond where one track ends (red circle), the frond where the next track begins (blue square), and the same well zoomed out showing both markers, with track identifiers labelled. The decision recorded for each is shown at right with the gap in frames and the separation in pixels. Two pairs were confirmed as the same frond; one was skipped because no frond lay beneath the marker. (B) Five fronds whose tracks fragmented during the 1,664-frame petri dish series (138.7 h). Pale bars are the raw btrack tracks, broken at each split; red marks the joins confirmed by the user; the dark bar beneath is the single identity obtained after correction. Across the full series, 6 pairs were linked automatically and 43 were shown for confirmation (42 confirmed, none rejected, one skipped), reducing identity switches from 35 to 11 (0.11% to 0.03%) and resolving 367 raw tracks into 319 fronds**.**

**Figure S14. Segmentation performance across a density gradient.** Sparse (left, external, 24 fronds), medium (center, external, 126 fronds), dense (right, in-domain petri dish, densely packed). Each panel: raw image, ground truth, best-performing model's prediction. Sparse and medium: microfluidics model, threshold recalibrated for the external domain (pooled body Dice 69.9%, IoU 53.7%). Dense: petri-dish 512x512 model (body Dice 88.5%, IoU 79.3%).

# 3. Supplementary Tables

**Table S1. Imaging System Specifications.** Technical specifications of the imaging system and experimental parameters used for time-lapse data acquisition.

| **Parameter** | **Microfluidic Chip** | **Petri Dish** |
| --- | --- | --- |
| Culture system | Custom microfluidic chip | 6-well Petri dish |
| Image format | JPEG | JPEG |
| Video compilation | 512 × 512 pixels | 512 × 512 pixels |
| Analysis resolution | 0.00111 mm²/pixel | Platform-specific |
| Pixel-to-area conversion | 20 min/frame | 5 min/frame |
| Pixel-to-length conversion | 338 | 300 |
| Time-lapse interval | 112.7 h (~4.7 days) | 140 h (~ 6 days) |
| Total frames | Custom microfluidic chip | 6-well Petri dish |
| Experiment duration | JPEG | JPEG |
| Culture system | 512 × 512 pixels | 512 × 512 pixels |
| Image format | 0.00111 mm²/pixel | Platform-specific |

**Table S2. U-Net Model Performance Metrics. Training** configuration and performance metrics for the U-Net segmentation model, including hyperparameters and final validation scores.

| **Metric** | **Value** | **Notes** |
| --- | --- | --- |
| Dice coefficient (validation) | 0.6767 | Best performance |
| IoU | 0.5166 | Measured on validation set |
| Training epochs | 470 | Total trained |
| Architecture | U-Net | Encoder-decoder |
| Input/Output size | 512×512×3 / 512×512×1 | RGB → binary mask |
| Loss function | Dice loss |  |
| Optimizer | Adam | LR: 1×10⁻⁴ initial |
| LR scheduler | ReduceLROnPlateau | Factor=0.5, patience=20 |
| Batch size / Dropout | 8 / 0.2 |  |
| Dice coefficient (independent, 512x512 model) | 0.908 +/- 0.006 | 10 frames, 324 fronds, 24-59 h after training |
| IoU (independent, 512x512 model) | 0.832 +/- 0.010 | Full resolution, 1600 x 1200 |
| Detection recall (independent, 512x512) | 85.8% | 278 of 324 fronds, IoU >= 0.5 |
| Detection precision (independent, 512x512) | 99.3% | 278 of 280 predictions |
| Dice coefficient (independent, 512x512 model) | 0.865 +/- 0.015 | Same 10 frames, original model |
| IoU (independent, 512x512 model) | 0.762 +/- 0.023 | Full resolution, 1600 x 1200 |
| Detection recall (independent, 512x512) | 77.5% | 251 of 324 fronds, IoU >= 0.5 |
| Detection precision (independent, 512x512) | 96.2% | 251 of 261 predictions |

**Table S3. Tracking and Detection Statistics.** Summary statistics from tracking analysis for both experimental platforms. Higher track counts in microfluidic experiments reflect longer duration and higher frond density.

| **Metric** | **Microfluidic Chip** | **Petri Dish** |
| --- | --- | --- |
| Total Frames Analyzed | 338 | 300 |
| Total Detections | 12,824 | 3,583 |
| Unique Frond Tracks | 192 | 35 |
| Average Track Length | 66.77 frames | 102.6 frames |
| Median Track Length | 14 frames | 82 frames |
| Longest Track | 340 frames | 300 frames |
| Budding Events Detected | 24 | 8 |
| Maximum Generation | 2 | 2 |
| Experiment Duration (hours) | 112.67 | 140 |
| Experiment Duration (days) | 4.69 | 6 |
| Time Resolution | 20 minutes/frame | 5 minutes/frame |
| Tracking Algorithm | btrack (Bayesian tracking) | btrack (Bayesian tracking) |
| Tracking Configuration | cell_config.json | cell_config.json |
| Maximum Search Radius | 50 pixels | 50 pixels |
| ID switch rate | 0.57% (78 / 13,757) | 0.11% (35 / 32,111) |
| Track fragmentation rate | 10.1% (22 / 218) | 11.7% (43 / 367) |
| ID switches Q1 / Q2 / Q3 / Q4 | 0.49% / 0.56% / 0.48% / 0.67% | 0.00% / 0.22% / 0.09% / 0.10% |

**Table S4. Per-Generation Morphological Statistics.** Morphological measurements by generation for both platforms. N (det.) = total detections across all frames; N (fronds) = unique individuals tracked. Note: absolute area differences between platforms reflect different pixel calibration factors for each imaging setup.

**Microfluidic Chip**

| **Gen** | **N (det.)** | **N (fronds)** | **Mean (mm²)** | **SD (mm²)** | **Median (mm²)** | **Range (mm²)** |
| --- | --- | --- | --- | --- | --- | --- |
| 0 | 12,498 | 161 | 0.096 | 0.03 | 0.039 | 0.001 - 0.216 |
| 1 | 290 | 92 | 0.05 | 0.02 | 0.013 | 0.001 - 0.048 |
| 2 | 32 | 15 | 0.04 | 0.01 | 0.011 | 0.002 - 0.025 |
|  |  |  |  |  |  |  |

**Petri Dish**

| **Gen** | **N (det.)** | **N (fronds)** | **Mean (mm²)** | **SD (mm²)** | **Median (mm²)** | **Range (mm²)** |
| --- | --- | --- | --- | --- | --- | --- |
| 0 | 2,985 | 162 | 2.68 | 0.97 | 2.443 | 0.69 - 3.97 |
| 1 | 510 | 63 | 1.45 | 1.01 | 1.647 | 0.61 - 3.90 |
| 2 | 86 | 4 | 3.23 | 0.67 | 3.053 | 2.91 - 4.17 |

**Table S5. Parent-Child Lineage Relationships.** Sample of inferred parent-child relationships from both platforms. Spatial distances typically <1 pixel for microfluidic experiments and <15 pixels in Petri dish experiments validate accurate budding detection. Frond 55 in the Petri dish experiment is itself a daughter of Frond 6, demonstrating successful multi-generational tracking. Complete data are available in supplementary files.

**Microfluidic Chip (24 total budding events)**

| **Parent ID** | **Child ID** | **Budding Frame** | **Spatial Distance (px)** | **Parent Gen** | **Child Gen** |
| --- | --- | --- | --- | --- | --- |
| 38.000 | 172.000 | 205 | 0.000 | 0.000 | 1.000 |
| 44.000 | 77.000 | 97 | 15.236 | 0.000 | 1.000 |
| 55.000 | 91.000 | 110 | 0.500 | 0.000 | 1.000 |
| 75.000 | 93.000 | 112 | 9.956 | 0.000 | 1.000 |
| 81.000 | 85.000 | 103 | 0.667 | 0.000 | 1.000 |
| 95.000 | 97.000 | 118 | 0.167 | 0.000 | 1.000 |
| 99.000 | 109.000 | 129 | 1.000 | 0.000 | 1.000 |
| 106.000 | 116.000 | 143 | 0.500 | 0.000 | 1.000 |
| 110.000 | 288.000 | 315 | 0.000 | 0.000 | 1.000 |
| 116.000 | 119.000 | 149 | 0.500 | 1.000 | 2.000 |
| 126.000 | 127.000 | 166 | 0.498 | 0.000 | 1.000 |
| 127.000 | 130.000 | 169 | 0.008 | 1.000 | 2.000 |
| 204.000 | 243.000 | 260 | 3.500 | 0.000 | 1.000 |
| 209.000 | 275.000 | 307 | 0.000 | 0.000 | 1.000 |
|  |  |  |  |  |  |

**Petri Dish (8 total budding events)**

| **Parent ID** | **Child ID** | **Budding Frame** | **Spatial Distance (px)** | **Parent Gen** | **Child Gen** |
| --- | --- | --- | --- | --- | --- |
| 38.000 | 172.000 | 205 | 0.000 | 0.000 | 1.000 |
| 44.000 | 77.000 | 97 | 15.236 | 0.000 | 1.000 |
| 55.000 | 91.000 | 110 | 0.500 | 0.000 | 1.000 |
| 75.000 | 93.000 | 112 | 9.956 | 0.000 | 1.000 |
| 81.000 | 85.000 | 103 | 0.667 | 0.000 | 1.000 |
| 95.000 | 97.000 | 118 | 0.167 | 0.000 | 1.000 |

**Table S6. Population Growth Model Parameters.** Fitted exponential growth model parameters. Model: N(t) = N₀ × exp(r × t). Microfluidic chip parameters were fitted to the aggregate population across all channels [95% CI]. Petri dish parameters were fitted to a single representative well (cluster 6) over the full 139-hour experiment (± SE). The faster growth rate (shorter doubling time) in the Petri dish likely reflects reduced spatial constraint compared to the microfluidic channels, where a substantial fraction of plants did not bud during the observation window. Observed population counts may underrepresent actual values because fronds moved out of the observation area during the experiment.

| **Parameter** | **Microfluidic Chip** | **Petri Dish** |
| --- | --- | --- |
| Initial Population (N₀) | 1 fronds | 2 fronds |
| Growth Rate r (hr⁻¹) | 0.00863 | 0.00778 |
| Doubling Time Td (hours) | 80.3 | 85.9 |
| R² (Goodness of Fit) | 0.5077 | 0.8148 |
| Total Duration (hours) | 112.33 | 139 |
| Initial Population (Observed) | 1 fronds | 2 fronds |
| Final Population (Observed) | 2 fronds | 6 fronds |
| Data Smoothing | 15-frame (5-hour window) | 15-frame (5-hour window) |

# 4. Supplementary Data

All data and code are available for reproducibility via:

<https://github.com/SamOliveiraLab/Deepweed>

Deepweed source code is freely available at: <https://github.com/SamOliveiraLab/Deepweed>

and archived at: <https://doi.org/10.5281/zenodo.18844282>

Datasets are archived at: <https://doi.org/10.5281/zenodo.18843917>

**Microfluidics Chip Analysis:**

Trained model: best_model_0707.pt

Architecture: unet_model_class.py

Training notebook: Duckweed_Paper_Analysis_v5.ipynb

Tracking config: cell_config.json

**Petri dish Analysis:**

Trained model: best_instance_unet.pt

Architecture: unet_model_class.py

Training notebook: Duckweed_Paper_Analysis_v5.ipynb

# 5. Supplementary Videos

Plant segmentation and tracking examples. Example time series videos are available as **Supplementary Videos S1-4**.

**Supplementary Videos S1**. Demonstratiion of the tracking performance for all petri dishes (raw data vs. segmented masks).

**Supplementary Videos S2**. Demonstratiion of the tracking performance for a single petri dishes (cluster 6) among all dishes (raw data vs. segmented masks).

**Supplementary Videos S3**. Demonstratiion of the tracking performance for all linear trenches of the microfluidic chip (raw data vs. segmented masks).

**Supplementary Videos S4**. Demonstratiion of the tracking performance for a single linear trench (cluster 7) of the microfluidic chip (raw data vs. segmented masks).
