# Supplementary material for: Deepweed: automated individual segmentation and tracking for duckweed lineage analysis
Source: Bioinformatics. 2026 Oct 16;42(10):btag779. doi: 10.1093/bioinformatics/btag779 (PMC13713196; doi:10.1093/bioinformatics/btag779)
Supplement: Supplementary file 1 [file btag779_supplementary_data.zip › Oliveira2026_SI_Tables_Rev2.pdf]

Supplementary Tables for "Deepweed: Automated Individual Segmentation and Tracking for Duckweed Lineage Analysis"

Tolulope Oyeniran[1,+], Bukola Akindipe[1,+], Tasmia Islam[2], Joshua D. Scheel[4], Eric A. Josephs[2,3], and Samuel M.D. Oliveira[1,*]

[1]Department of Nanoengineering, North Carolina Agricultural and Technical State University, Greensboro, NC, USA 27401,[2]Department of Nanoscience, University of North Carolina at Greensboro, NC, USA 27403,[3]Department of Biomedical Engineering, Stony Brook University, 100 Nicolls Road, Stony Brook, NY, USA 11794, [4]Department of Biomedical Engineering, University of Texas at Austin, Austin, TX, USA 78712

[+]Authors contributed equally
*Corresponding author: smdoliveira@ncat.edu

Supplementary Tables

Table S1. Imaging System Specifications. Technical specifications of the imaging system and experimental parameters used for time-lapse data acquisition.

**Parameter**	**Microfluidic Chip**	**Petri Dish**
Culture system	Custom microfluidic chip	6-well Petri dish
Image format	JPEG	JPEG
Video compilation	256 × 256 pixels	256 × 256 pixels
Analysis resolution	0.00111 mm²/pixel	Platform-specific
Pixel-to-area conversion	20 min/frame	5 min/frame
Pixel-to-length conversion	338	300
Time-lapse interval	112.7 h (~4.7 days)	140 h (~ 6 days)
Total frames	Custom microfluidic chip	6-well Petri dish
Experiment duration	JPEG	JPEG
Culture system	256 × 256 pixels	256 × 256 pixels
Image format	0.00111 mm²/pixel	Platform-specific

Table S2. U-Net Model Performance Metrics. Training configuration and performance metrics for the U-Net segmentation model, including hyperparameters and final validation scores.

Metric	Value	Notes
Dice coefficient (validation)	0.8720	Best performance
IoU	0.7730	From Dice
Training epochs	470	Total trained
Architecture	U-Net	Encoder-decoder
Input/Output size	256×256×3 / 256×256×1	RGB → binary mask
Loss function	Dice loss	
Optimizer	Adam	LR: 1×10^{-4} initial
LR scheduler	ReduceLROnPlateau	Factor=0.5, patience=20
Batch size / Dropout	8 / 0.2	
Dice coefficient (independent, 512x512 model)	0.908 +/- 0.006	10 frames, 324 fronds, 24-59 h after training
IoU (independent, 512x512 model)	0.832 +/- 0.010	Full resolution, 1600 x 1200
Detection recall (independent, 512x512)	85.8%	278 of 324 fronds, IoU >= 0.5
Detection precision (independent, 512x512)	99.3%	278 of 280 predictions
Dice coefficient (independent, 256x256 model)	0.865 +/- 0.015	Same 10 frames, original model
IoU (independent, 256x256 model)	0.762 +/- 0.023	Full resolution, 1600 x 1200
Detection recall (independent, 256x256)	77.5%	251 of 324 fronds, IoU >= 0.5
Detection precision (independent, 256x256)	96.2%	251 of 261 predictions

Table S3. Tracking and Detection Statistics. Summary statistics from tracking analysis for both experimental platforms. Higher track counts in microfluidic experiments reflect longer duration and higher frond density.

Metric	Microfluidic Chip	Petri Dish
Total Frames Analyzed	338	300
Total Detections	12,824	3,583
Unique Cell Tracks	192	35
Average Track Length	66.77 frames	102.6 frames
Median Track Length	14 frames	82 frames
Longest Track	340 frames	300 frames
Division Events Detected	24	8
Maximum Generation	2	2
Experiment Duration (hours)	112.67	140
Experiment Duration (days)	4.69	6
Time Resolution	20 minutes/frame	5 minutes/frame
Tracking Algorithm	btrack (Bayesian tracking)	btrack (Bayesian tracking)
Tracking Configuration	cell_config.json	cell_config.json
Maximum Search Radius	50 pixels	50 pixels
ID switch rate	0.57% (78 / 13,757)	0.11% (35 / 32,111)
Track fragmentation rate	10.1% (22 / 218)	11.7% (43 / 367)
ID switches Q1 / Q2 / Q3 / Q4	0.49% / 0.56% / 0.48% / 0.67%	0.00% / 0.22% / 0.09% / 0.10%

Table S4. Population Growth Model Parameters. Fitted exponential growth model parameters. Model: $N(t) = N_0 \times \exp(r \times t)$. Microfluidic chip parameters were fitted to the aggregate population across all channels [95% CI]. Petri dish parameters were fitted to a single representative well (cluster 6) over the full 139-hour experiment (± SE). The faster growth rate (shorter doubling time) in the Petri dish likely reflects reduced spatial constraint compared to the microfluidic channels, where a substantial fraction of plants did not bud during the observation window. Observed population counts may underrepresent actual values because fronds moved out of the observation area during the experiment.

Parameter	Microfluidic Chip	Petri Dish
Initial Population (N_0)	1 fronds	2 fronds
Growth Rate r (hr^{-1})	0.00863	0.00778
Doubling Time Td (hours)	80.3	85.9
R^2 (Goodness of Fit)	0.5077	0.8148
Total Duration (hours)	112.33	139
Initial Population (Observed)	1 fronds	2 fronds
Final Population (Observed)	2 fronds	6 fronds
Data Smoothing	15-frame (5-hour window)	15-frame (5-hour window)

Table S5. Per-Generation Morphological Statistics. Morphological measurements by generation for both platforms. N (det.) = total detections across all frames; N (fronds) = unique individuals tracked. Note: absolute area differences between platforms reflect different pixel calibration factors for each imaging setup.

Microfluidic Chip

Gen	N (det.)	N (fronds)	Mean (mm^2)	SD (mm^2)	Median (mm^2)	Range (mm^2)
0	12,498	161	0.096	0.03	0.039	0.001 - 0.216
1	290	92	0.05	0.02	0.013	0.001 - 0.048
2	32	15	0.04	0.01	0.011	0.002 - 0.025

Petri Dish

Gen	N (det.)	N (fronds)	Mean (mm^2)	SD (mm^2)	Median (mm^2)	Range (mm^2)
0	2,985	162	2.68	0.97	2.443	0.69 - 3.97
1	510	63	1.45	1.01	1.647	0.61 - 3.90
2	86	4	3.23	0.67	3.053	2.91 - 4.17

Table S6. Parent-Child Lineage Relationships. Sample of inferred parent-child relationships from both platforms. Spatial distances typically <1 pixel for microfluidic experiments and <15 pixels in Petri dish experiments validate accurate budding detection. Cell 55 in the Petri dish experiment is itself a daughter of Cell 6, demonstrating successful multi-generational tracking. Complete data available in supplementary files.

Microfluidic Chip (24 total division events)

Parent ID	Child ID	Division Frame	Spatial Distance (px)	Parent Gen	Child Gen
38.000	172.000	205	0.000	0.000	1.000
44.000	77.000	97	15.236	0.000	1.000
55.000	91.000	110	0.500	0.000	1.000
75.000	93.000	112	9.956	0.000	1.000
81.000	85.000	103	0.667	0.000	1.000
95.000	97.000	118	0.167	0.000	1.000
99.000	109.000	129	1.000	0.000	1.000
106.000	116.000	143	0.500	0.000	1.000
110.000	288.000	315	0.000	0.000	1.000
116.000	119.000	149	0.500	1.000	2.000
126.000	127.000	166	0.498	0.000	1.000
127.000	130.000	169	0.008	1.000	2.000
204.000	243.000	260	3.500	0.000	1.000
209.000	275.000	307	0.000	0.000	1.000

Petri Dish (8 total division events)

Parent ID	Child ID	Division Frame	Spatial Distance (px)	Parent Gen	Child Gen
38.000	172.000	205	0.000	0.000	1.000
44.000	77.000	97	15.236	0.000	1.000
55.000	91.000	110	0.500	0.000	1.000
75.000	93.000	112	9.956	0.000	1.000
81.000	85.000	103	0.667	0.000	1.000
95.000	97.000	118	0.167	0.000	1.000
